# Amniotic Membrane and Stem Cells Can Improve the Immunohistochemical Profile of Achilles Tendons in Injured Rats

**DOI:** 10.3390/biomedicines13123018

**Published:** 2025-12-09

**Authors:** Rosangela Alquieri Fedato, Guilherme Vieira Cavalcante Da Silva, Lúcia De Noronha, Seigo Nagashima, Ana Paula Camargo Martins, Márcia Olandoski, Ricardo Aurino De Pinho, Aline Luri Takejima, Rossana Baggio Simeoni, Júlio Cesar Francisco, Luiz César Guarita-Souza

**Affiliations:** 1 Department of Cellular and Molecular Biology and Experimental Pathology, Pontifical Catholic University of Paraná, Curitiba 80215-901, PR, Brazil; 2 Department of Bioestatistics, Pontifical Catholic University of Paraná, Curitiba 80215-901, PR, Brazil

**Keywords:** achilles tendonitis, tendonitis treatment, cell therapies, IL-4, IL-13

## Abstract

Disorders of the Achilles tendon are common and have a major socio-economic impact. Current treatments (drugs, physiotherapy, and surgery) do not provide lasting relief, leading to chronicity and recurrence. In this context, experimental studies on regenerative therapies, such as stem cells, and natural and synthetic membranes, have shown promising results in the treatment of tendon lesions. **Background/Objectives**: The present study analyzes the response of tissue to a combination of bone marrow mononuclear cells (BMMCs) and human decellularized amniotic membrane (AM) for the treatment of Achilles tendon lesions in rats. **Methods**: Forty male Wistar rats were randomized into four treatment groups: SC (stem cells), AM (amniotic membrane), SC + AM (stem cells + amniotic membrane), and C (control). All underwent Achilles tendon sectioning and tenorrhaphy. In the AM and SC + AM groups, the amniotic membrane was sutured over the lesion after the tendon was sutured; in the SC and SC + AM groups, 2 mL of autologous blood from the iliac crest containing BMMCs was applied around the lesion. Animals in Group C received only 2 mL of 0.9% saline around the lesion. After four weeks, the animals were euthanized, and the tendons were sent for histological analysis (*Picrosirius Red*) and immunohistochemistry (IL-6, IL-4, and IL-13). **Results**: Analysis of type I and type III collagen fibers showed no differences between groups. However, the SC + AM group showed the highest expressions of IL-4 and IL-13. **Conclusions**: IL-4 and IL-13 are cytokines known to be associated with tissue repair and organization. This suggests that the therapy associated with SC and AM is potentially beneficial in the treatment of injured Achilles tendons. However, further studies are necessary to clarify the benefits of this treatment for the function and biomechanical properties of the tendon and prove whether this association could represent a combined Advanced Therapy Medicinal Product (cATMP). Such a product would contain SC and a biological membrane, providing a mechanical structure for the injured tendon and active biological cells. Another possible medical approach could be immunobiological drugs targeting IL-4 and IL-13.

## 1. Introduction

Tendon disorders are common in clinical practice and have low healing potential in both acute and chronic injuries [1,2]. Current treatments, based on anti-inflammatory drugs, rehabilitation programs, and surgery, fail to provide lasting and significant relief, and there is no therapeutic consensus or modality that is considered a gold standard [3,4].

Within this context, advanced therapies have emerged as promising options [5]. Among cell therapies, bone marrow mononuclear stem cells (BMMCs) are a type of stem cell (SC) that can differentiate into tenocytes and induce the formation of linearly arranged type I collagen, as well as an increase in resistance to stress modulus, tension, and deformation, improving the biomechanical characteristics of tendons [6,7,8].

Synthetic membranes, including silk or natural membranes, as the latter of which includes amnion, have the primary function of providing a structural framework. Amnion, a connective membrane associated with the placenta, is known for its anti-inflammatory and antimicrobial properties and low antigenicity when decellularized [9]. In controlled studies, amniotic membrane fragments placed in injured tendons are associated with better organization of type I collagen compared to the control group [10,11,12].

Considering the inflammatory pathways involved in tendon injury and repair, several cytokines have been investigated as potential therapeutic targets to modulate excessive or dysregulated inflammation. In this context, cytokines such as IL-4 and IL-13 are of particular interest due to their roles in regulating immune responses and promoting tissue remodeling. Therefore, understanding their activity in tendon healing may help guide future strategies aimed at improving the biological environment during repair [13,14]. Regarding the molecular process of tissue repair in tendinitis [15], interleukin-6 (IL-6) is an acute-phase pro-inflammatory cytokine; however, it can persist late in inflammatory processes, assuming an immunomodulatory function, and is related to tendon healing [16]. In canine and murine models, the expression of the interleukin-6 gene is increased in animals subjected to tendon injury. This is similar to how, in humans, pertitendinous infiltration of IL-6 stimulates collagen synthesis [17].

Interleukin-4 (IL-4) is also involved in collagen production by fibroblasts. It forms negative feedback with IL-6 and interferon-gamma, antagonizing their initial pro-inflammatory response, and appears in later inflammatory processes. It controls the initial inflammatory process for effective tissue repair [18]. IL-4 does not act directly in the collagen formation process but presents positive feedback with interleukin-13 (IL-13) [19].

IL-13, closely related to IL-4, regulates the breakdown of type III collagen (disorganized, initial) and the consequent deposition of type I collagen (mature and linear) through the subsequent activation of extracellular matrix metalloproteinases (MMPs). One of the main ways in which IL-13 promotes collagen synthesis is through the activation and amplification of TGF-β (tumor growth factor beta) signaling, which stimulates fibroblasts. MMP-9, mediated by IL-13, cleaves latent TGF-β into active TGF-β and stimulates its expression. However, fibroblasts also respond to the direct action of IL-13 and IL-4, independently of TGF-β’s action [20].

Since mesenchymal stem cells are capable of remodeling injured tendons and increasing biomechanical properties (maximum stress, modulus, and strain), tenocyte number, and number and quality of mature collagen fibers [5,6,7,8], amnion-derived membranes and cells have been reported to have the capacity for multipotent differentiation [9,10,11,12]. Both show complementary therapeutic effects, justifying the study of their combined use in the treatment of inflammatory tendinopathies, for which current therapies have a high failure rate [3,4].

Therefore, this study aimed to assess whether a combination of BMMCs and AM induces a synergistic response in the treatment of ruptured Achilles tendons in rats.

## 2. Materials and Methods

Following approval by the Ethics Committee of the Victor Ferreira do Amaral Maternity Hospital (registration 01238, 2 August 2018, Curitiba, Paraná, Brazil), placentas were collected from two parturient women. The animal experiment was approved by the Animal Care and Use Committee of the Pontifical Catholic University of Paraná (CEUA-Pr 01637, 26 June 2019) and international animal welfare standards were followed (ARRIVE guidelines). Forty male Wistar rats, weighing an average of 350 g, were used.

### 2.1. Experimental Lesion Model

**Figure 1:**
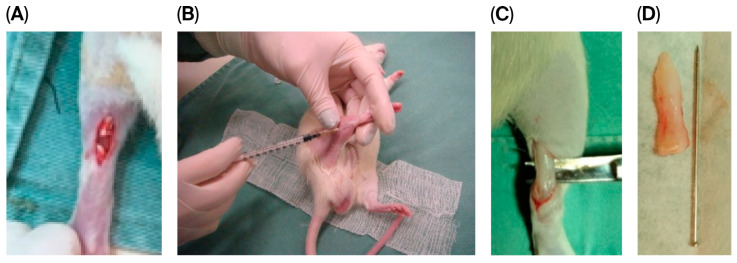
Experimental steps of the animal model lesion. (**A**): Achilles tendon following skin incision and tenotomy. (**B**): Percutaneous application of stem cells/0.9% saline solution. (**C**): Intraoperative image of the tendon at 4 weeks, at the time of euthanasia, and tendon resection. (**D**): Resected tendon specimen.

The rats were anesthetized intraperitoneally with 5% ketamine hydrochloride (Vetanarcol^TM^, Konig do Brazil Ltd., Mairinque, Brazil) at a dose of 80 mg/kg and combined with 2% xylazine hydrochloride (Rompun^TM^, Bayer S.A., São Paulo, Brazil) at a dose of 10 mg/kg. Once anesthesia was achieved, as verified by pupillary reflex and muscle relaxation, the surgical site was shaved, and tenotomy was performed, as demonstrated in Figure 1A.

In brief, a linear skin incision was made in the right hind leg of each rat; the Achilles tendon was individualized, bisected completely at the middle third, and sutured with 5–0 polypropylene (PolipropypointPoint Suture, Mairinque, Brazil).

After surgery, animals were randomized into four treatment groups (*n* = 10 each):

- SC group, BMMCs only;
- AM group, AM only;
- SC + AM group, BMMCs + AM;
- C group (control), 0.9% saline solution.

For animals in the AM and SC + AM groups, after the Achilles lesion had been bridged with polypropylene for 5–0 sutures, a decellularized amniotic membrane was fitted and sutured to the tendon along its edges with 4 stitches at its ends (as well as 5–0 polypropylene) The technique for obtaining, preparing, and storing the amniotic membrane is described below.

Skin closure was performed with 5–0 polypropylene, after which animals in the SC and SC + AM groups were percutaneously infiltrated with stem cells in the Achilles peritendon (Figure 1B), and animals in the C group were percutaneously infiltrated with 2 mL of 0.9% saline solution (the technique for stem cell isolation and preparation is also described below).

Four weeks after the induction of injury, the animals were euthanized according to the anesthesia protocol described above, and the tendons were resected and sent for collagen and immunohistochemical evaluation (Figure 1C,D).

### 2.2. Isolation and Preparation of Bone Marrow Mononuclear Cells

For rats in the SC and SC + AM groups, the procedure began with the collection of blood from their iliac crests to prepare and isolate stem cells according to the technique described by Boyum, namely separation by density gradient using Iscove’s Modified Dulbecco’s Media (IMDM) and density gradient separation solution (Ficoll-Hypaque—Ficoll^®^ Paque Plus GE17-1440-02, liquid, sterile, endotoxins < 0.12 EU/mL, density: 1.077 g/mL, pack of 6 × 100 mL, Cytiva, Marlborough, MA, USA) [21], as explained below.

To obtain the BMMCs, the right iliac crest of each animal was punctured with a 14-gauge needle, and approximately 2–3 mL of blood was aspirated into anticoagulant-containing tubes, isolated by Ficoll density gradient, and cultured in IMDM medium supplemented with antibiotics (penicillin and streptomycin 1%). After counting in a Neubauer chamber, the cells were resuspended in sterile PBS (1 × 10^5^ cells/μL) and injected into the site of the tendinous lesion using a syringe and insulin needle.

### 2.3. Amniotic Membrane Preparation

Fresh amniotic membrane (AM) was obtained after cesarean deliveries from human maternal donors with negative serologies for HIV, hepatitis B, hepatitis C, and syphilis.

After delivery of the placenta, blood clots were immediately removed by washing the placenta with phosphate-buffered saline solution (PBS) pH 7.2, containing 100 u/mL penicillin and 100 mg/mL streptomycin (Gibco^®^, Grand Island, NY, USA).

**Figure 2:**
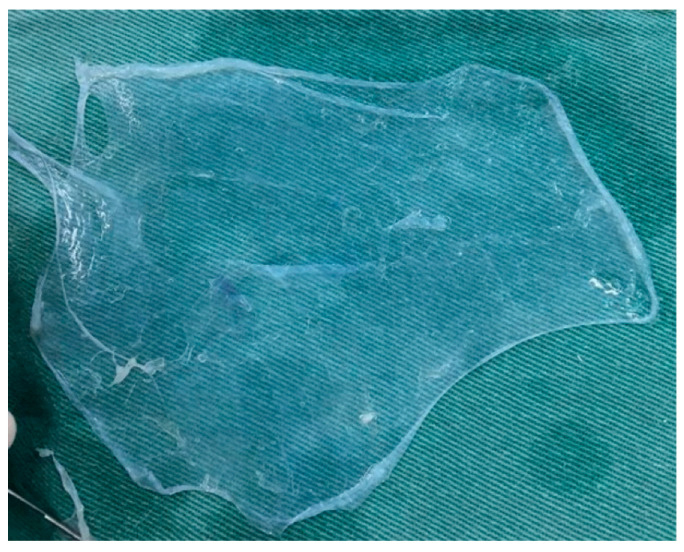
Final aspect of the amniotic membrane after preparation and decellularization.

Decellularization of the AM was performed by removing amniotic epithelial cells from the membrane using 0.01% sodium dodecyl sulfate and 0.01% sodium deoxycholate in PBS. Incubation was performed at a rotation speed of 100 rpm at 37 °C for 24 h in a class II BioSAFE biological safety cabinet (Veco^®^, Plainview, NY, USA). The AMs were then washed three times with PBS for further decellularization and preserved in PBS at 4 °C [22,23]; Figure 2 shows the final appearance of the decellularized AM.

### 2.4. Histological and Immunohistochemical Analysis

Histological sections were mounted for Picrosirius Red staining (Direct Red: Aldrich Chemical Company Inc., Milwaukee, WI, USA) to specifically characterize collagen fibers type I (red) and type III (green) under a circular polarization lens. Ten high-power field (HPF) generation was performed.

The immunohistochemistry technique was used to identify the immunoexpression of interleukin-4 (IL-4, PAS-25165, 1:200, Thermo Fisher Scientific, AB_2542665, Waltham, MA, USA), interleukin-6 (IL-6, MP5-2OF3, Thermo Fisher Scientific, AB_469216, Waltham, MA, USA) and interleukin-13 (IL-13, P130-E, 1:600, Thermo Fischer Scientific, AB_223471ABclonal, Manhattan Beach, CA, USA). Immunohistochemistry was carried out as follows: Primary antibodies were incubated in a humid chamber at 2–8 °C overnight. Subsequently, the secondary polymer (Reveal Polyvalent HRP-DAB Detection System, Spring Bioscience, Pleasanton, CA, USA) was applied to the sections for 25 min at room temperature. Visualization was achieved through exposure to the 2,3-diaminobenzidine (DAB) complex with the hydrogen peroxide substrate, allowing sufficient time for development of the brown chromogenic signal, followed by counterstaining with Harris’ hematoxylin. Specificity of the reaction was verified using a positive control tissue sample with known antibody immunoreactivity, which was processed in parallel with the test samples.

The slides were immunolabeled with anti-IL-4, anti-IL-6, and anti-IL-13 antibodies, scanned in an Axio Scan.Z1 slide scanner (Zeiss, Jena, Germany), and the analyzed images were generated with ZEN 2.3 Blue Edition software (Zeiss, Jena, Germany). We performed 30 HPF generation randomly using the software, with no interference from the investigator. In each HPF, areas of immunoexpression were measured using Image Pro-Plus software version 4.5 (Media Cybernetics, Rockville, MD, USA) and a semi-automated color segmentation method, in which the tissue area immunoexpression for each biomarker was artificially delimited and quantified.

Subsequently, the area, expressed in square micrometers (μm^2^), was divided by the respective total tissue area and represented as a percentage. Finally, the arithmetic mean value expression of each biomarker of interest was calculated in the HPFs of each sample. The results were organized in Microsoft Excel^®^ spreadsheets and analyzed in IBM SPSS Statistics for Windows, Version 29.0.

### 2.5. Statistical Analysis

The results were described as means, standard deviations, medians, and ranges (minimum–maximum). For comparisons among groups, the Kruskal–Wallis non-parametric test was applied. When significant, pairwise comparisons were performed using Dunn’s post hoc procedure and Bonferroni-adjusted *p*-values. The correlation between two quantitative variables was analyzed by estimating Spearman’s coefficients and assessing their significance. The normality of distribution of continuous variables was assessed using the Shapiro–Wilk test. *p*-values < 0.05 were deemed indicative of statistical significance, and analysis was performed using IBM^®^ SPSS Statistics v.20.0 software (IBM, Armonk, NY, USA).

## 3. Results

Of the forty animals included, three died after the tenotomy and tenorrhaphy procedure, including two in the SC + AM group and one in the control group. Thus, data from 37 rats were available for the final analysis: SC group *n* = 10; AM group *n* = 10; SC + AM group *n* = 8; and C group *n* = 9.

For the histological assessment of type I and III collagen fibers, Picrosirius Red staining was carried out, in which type I collagen stained red under illumination and type III collagen stained green.

**Figure 3:**
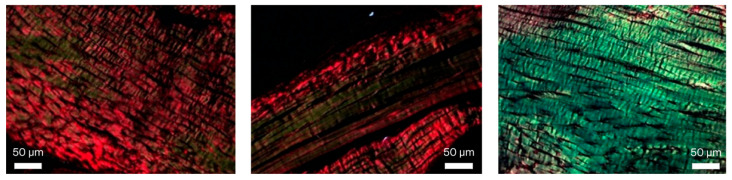
Picrosirius Red staining (400×), illustrating the variation in the ratio of type I to type III collagen fibers, ranging from a low proportion of type III (green) collagen (**left**), to moderate proportion (**middle**) and mainly type III collagen (**right**).

The figures below illustrate the variation in collagen composition among the samples, ranging from predominantly type I collagen (Figure 3, left) to a moderate presence of type I collagen (Figure 3, center) and mostly type III collagen (Figure 3, right). Since no statistically significant differences were observed in the proportions of type I and type III collagen between groups, these figures are presented for illustrative purposes only.

The slides were submitted to an automated process that assessed the percentage of type I collagen fibers and the percentage of type III collagen fibers, resulting in the values tabulated below (Table 1, Appendix A).

Thus, there was no statistically significant difference in the presence of type I and III collagen between the groups.

The immunohistochemistry technique was used to identify the immunoexpression of IL-6, IL-4, and IL-13; however, due to the loss of samples (slides in which the immunohistochemical staining was not fixed and, therefore, could not be analyzed), the final number for each group was broken down as follows in Table 2 and Appendix B.

**Figure 4:**
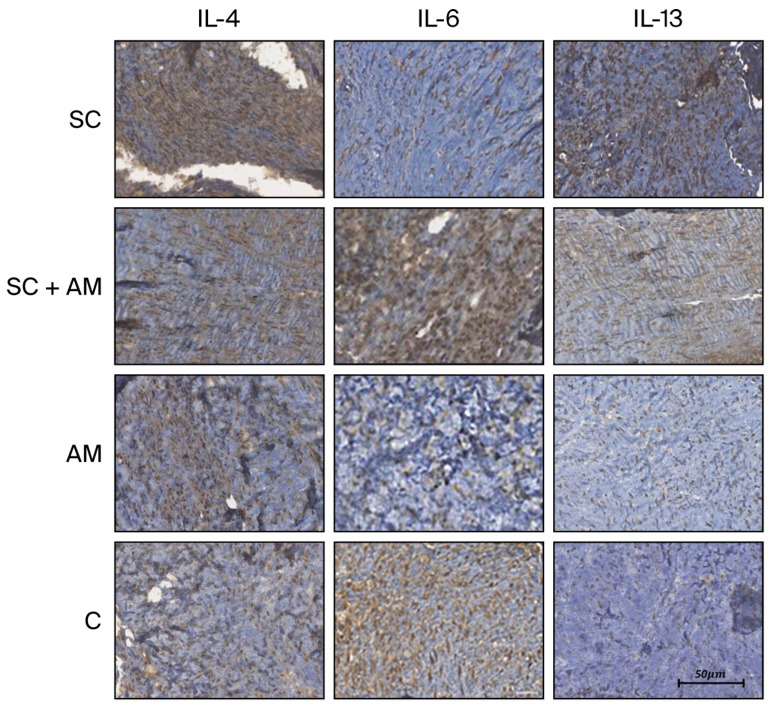
Images of immunohistochemical slides (×200) from each group based on the immunohistochemistry antibodies. Scale bar in 50 µm.

Figure 4 illustrates the slide analysis, highlighting the intensity of the tissue reaction induced by the antibodies in each group.

**Figure 5:**
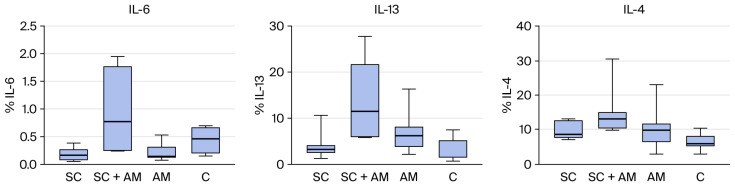
(**Left**): Distribution of the percentage of IL-6 among the groups. (**Middle**): Distribution of the percentage of IL-13 among the groups. (**Right**): Distribution of the percentage of IL-4 among the groups.

Photographs were generated from each slide and read automatically, generating the percentage of the area stained per total tissue area. The immunohistochemical reaction was assessed, resulting in the values tabulated below (Table 3) and presented in the subsequent graphs (Figure 5).

Significant differences were found for IL-13 and IL-4 between the groups. Therefore, comparisons were performed for two groups at a time, as shown in the table below (Table 4), which presents the *p*-values of these comparisons.

**Figure 6:**
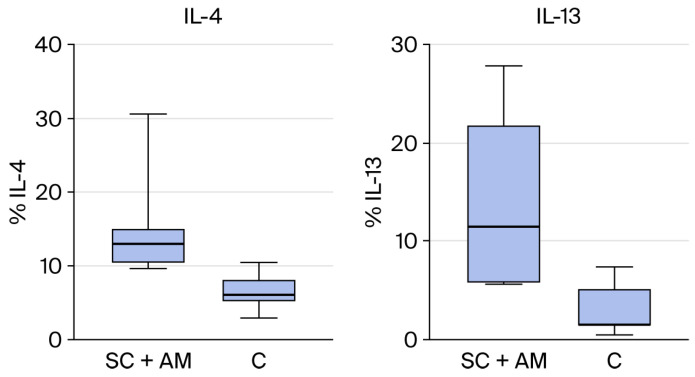
(**Right**): Statistical difference in the presence of IL-13 between groups SC + AM and C. (**Left**): Statistical difference in the presence of IL-4 between groups SC + AM and C.

From the results above, the groups that showed statistically significant differences are represented in the graphs below (IL-13 and IL-4 between C and SC + AM groups, Figure 6).

Finally, we performed a statistical analysis of the correlation between variables. Table 5 below contains the estimated Spearman correlation coefficients and the *p*-values of the statistical tests for significant data considering all groups.

The *p*-value for the correlation coefficient between IL-4 and collagen I and III is slightly above 0.05 (*p* = 0.0058), but Spearman’s coefficient demonstrates a strong biological correlation (Spearman’s coefficient above 0.6). This correlation is inverse or negative for Collagen I (−0.65) and direct or positive for Collagen III (0.65).

The *p*-value for the correlation coefficient between IL-13 and collagen I and III is slightly above 0.05, also (*p* = 0.0057), and Spearman’s coefficient demonstrates weak biological correlation (Spearman’s coefficient between 0.20 and 0.39). This correlation is inverse or negative for Collagen I (−0.35) and direct or positive for Collagen III (0.35).

**Figure 7:**
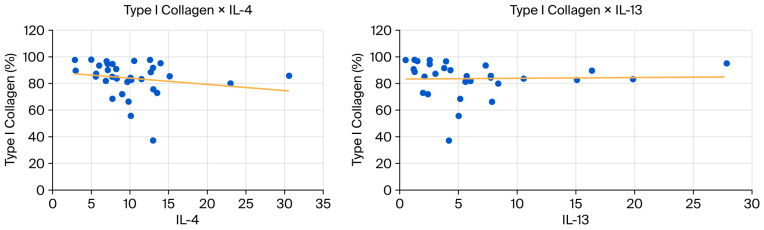
(**Left**): Scatter plot showing a negative correlation between type I collagen and IL-4. (**Right**): Scatter plot showing a negative correlation between collagen type I and IL-13.

**Figure 8:**
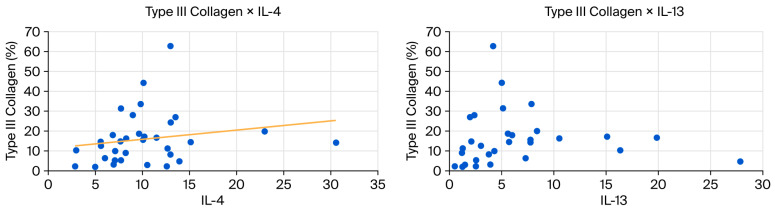
(**Left**): Scatter plot showing a positive correlation between type III collagen and IL-4. (**Right**): Scatter plot showing a positive correlation between collagen type III and IL-13.

These correlations are shown in the scatter diagrams below (Figure 7 and Figure 8).

## 4. Discussion

At the end of 4 weeks of treatment in this study, there was no significant difference across groups for the presence of type I and III collagen (assessed by Picrosirius Red) or immunoexpression of IL-6.

For the histological assessment of type I and III collagen fibers, the average percentage per group ranged from 80.3 to 88.4 for type I collagen and from 11.6 to 19.7 for type III collagen. Thus, all groups had a cellular matrix structure composed of >80% type I collagen fibers.

In our study, we observed that the levels of IL-6 did not show a significant difference among the groups analyzed. This finding can be attributed to the role of IL-6 as an acute-phase interleukin, which typically responds rapidly to inflammatory stimuli. As such, its correlation with collagen formation and tenocyte activity would only be present during the first few days following injury [24]. IL-6 peaks were observed between 3 days and up to 2 weeks after tendon injury; its elevated expression for longer periods is associated with dysfunction in the extracellular matrix by triggering a chronic positive feedback loop with interferon-gamma (IFN-g), which is an extracellular matrix-degrading interleukin [25,26]. Therefore, the lack of a significant difference in IL-6 may indicate a normal acute phase response rather than a pathological condition, emphasizing the need to consider the timing and phase of cytokine response when interpreting these results.

In contrast, IL-4 and IL-13, which did demonstrate significant differences, are classified as late-phase cytokines involved in the modulation of immune responses and the regulation of inflammation.

For these late-phase cytokines, IL-4 and IL-13 both showed significant differences between the groups; they were present at higher concentrations in the SC + AM group compared to the control group [27].

IL-4 provides negative feedback to IL-6 and regulates the initial inflammatory response, thereby promoting effective tissue repair through the stimulation of tenocyte differentiation and proliferation, supporting tissue repair and organization. The balance between IL-4 and other immune mediators, such as IL-13 and IL-10, is crucial for successful tendon regeneration [19,20,28,29,30].

The beneficial effects of IL-4 depend on both the timing and the context of the healing process. In certain situations, particularly during aging, an impaired immune response involving IL-4 and M2 macrophages can result in poor healing outcomes [20,28,31,32].

The significant increase in IL-4 observed in the SC + AM group suggests that tendon repair in this group is enhanced by the documented stimulatory effect of IL-4 on tenocytes.

IL-13, activated by IL-4, promotes tissue remodeling and collagen production by fibroblasts. Both interleukins correlate positively with the presence of type III collagen—the first collagen subtype to appear during tissue repair—while also promoting tenocyte differentiation and proliferation. IL-13 binds to receptors on tenocytes, stimulating genes that regulate the cell cycle and the production of proteins involved in tendon maintenance [19,28,33].

The correlations presented in Table 5 suggest biologically meaningful relationships between Th2 (IL-4 and IL-13)cytokines and collagen composition, despite the slightly superior *p* value (*p* = 0.0057 and *p* = 0.0058, respectively). In the MA group, IL-4 shows strong correlations with both collagen types: a strong negative association with collagen I (ρ = −0.65) and a strong positive association with collagen III (ρ = 0.65), with *p* = 0.058 for both. Although these values fall just above the significance threshold, the magnitude and consistency of the correlations indicate a plausible biological effect, likely underdetected due to the small sample size (*n* = 9). This pattern supports the known role of IL-4 in promoting a shift toward a more immature fibroblastic phenotype and favoring collagen III deposition over collagen I.

In the full dataset, IL-13 exhibits weaker correlations with the same collagen types (ρ = −0.35 for collagen I and ρ = 0.35 for collagen III; *p* = 0.057), but the directionality mirrors that observed for IL-4. This concordance reinforces the hypothesis that Th2-driven pathways may influence extracellular matrix remodeling by promoting a higher proportion of collagen III.

Overall, even though the associations did not reach statistical significance, the strong correlations in the MA group and the consistent trends across analyses suggest a potential Th2-mediated shift in collagen composition. Larger studies are needed to confirm these tendencies and clarify their functional relevance.

In the present study, the group treated with SC + AM showed increased levels of IL-13, corresponding to elevated levels of its upstream regulator, IL-4. Both interleukins correlated positively with type III collagen, confirming their involvement in the tendon regeneration pathway and further suggesting that this treatment facilitated repair.

The absence of histological differences across groups does not necessarily represent homogeneity, but indicates a moment when the repair process had not yet progressed far enough to exert morphologically visible differences. Considering the between-group differences in the inflammatory markers observed, it is likely that, if we were to continue the study and carry out a later histological assessment, we would have found histological differences as well.

This is further corroborated by a previous study by our group, in which we assessed the biomechanical strength and histology of Achilles tendons in rats treated with stem cells and platelet-rich plasma. During this study, despite there being no histological differences or changes in the presence of type I and III collagen between the groups, the platelet-rich plasma group showed better biomechanics, implicating molecular mechanisms in the repair process [34].

During tendon healing, IL-4 acts as a signal for IL-13, an effector cytokine that stimulates fibroblast production. The production of type III collagen is initially increased, which over time can mature into type I collagen, further organizing the extracellular matrix. This correlation was also observed in this study.

In the process of tissue repair, the amniotic membrane provides a collagenous support framework and facilitates the adhesion of growth factors, promoting healing in various types of lesions, including corneal lesions, venous ulcers, and skin burns [35,36]. The combination of amniotic membrane and mesenchymal stem cells has already proved effective in healing full-thickness skin defects in rats, which is a finding that encouraged us to test this combination in the treatment of tendon injury [37].

These therapies have potential use in clinical practice, as current biological treatments lack evidence of beneficial effects in Achilles tendonitis.

Beyond stem cells and amniotic-derived products, several other biological therapies have been investigated for Achilles tendinopathy, although the evidence remains inconsistent. Platelet-rich plasma (PRP) has been one of the most widely studied options; however, recent high-quality meta-analyses and randomized trials have failed to demonstrate clinically meaningful benefits over the placebo for midportion Achilles tendinopathy or acute ruptures, highlighting considerable heterogeneity in PRP formulations and application protocols [38,39,40]. Autologous tenocyte implantation (ATI) has shown promising early results, with improved pain and VISA-A scores in small prospective cohorts, but evidence remains limited by small sample sizes and lack of controlled trials [41]. More recently, extracellular vesicles derived from mesenchymal stromal cells have emerged as a cell-free regenerative therapy, demonstrating potent immunomodulatory and pro-tenogenic effects in preclinical tendon injury models. However, no clinical trials are yet available for humans with Achilles disease [42]. Growth-factor-based interventions—such as FGF-2, PDGF, and TGF-β3—have shown the capacity to enhance tenocyte proliferation and matrix organization in experimental models, yet translation into clinical practice has been slow due to safety concerns, short half-life, and delivery challenges [43]. Collagen scaffolds, either alone or combined with bioactive molecules, have also been explored to support tendon regeneration, but consensus regarding their effectiveness is still lacking [44]. Overall, while several biological therapies show theoretical and preclinical potential, robust clinical evidence supporting their routine use in Achilles tendinopathy is still insufficient, reinforcing the need for well-designed studies and standardized protocols.

Continued study of inflammatory pathways should identify potential targets for the specific and effective treatment of inflammatory and other chronic diseases, just as blocking IL-21 is effective in controlling rheumatoid arthritis [35].

This study presents some limitations. The use of an animal model may be associated with physiological and anatomical differences between rats and humans, which can limit the direct extrapolation of the results.

Current findings are also restricted to histological and immunohistochemical outcomes, which reflect molecular and structural aspects of healing but do not allow us to conclude whether these changes translate into improved biomechanical performance.

Moreover, the healing process in rodents tends to occur more rapidly than in humans. In addition, given the restricted sample size, the findings of this study should be considered preliminary.

We also emphasize that future studies incorporating standardized biomechanical testing are essential to validate the functional significance of the biological improvements observed in this study.

## 5. Conclusions

The molecular mechanisms underlying the pathogenesis of tendinopathies precede histological or morphological changes, making them a promising target for therapeutic intervention, as demonstrated in diseases such as rheumatoid arthritis.

In chronic tendinopathy, low levels of IL-4 and IL-13 are associated with persistent inflammation and impaired tissue repair. In contrast, rats treated with stem cells and amniotic membrane exhibited higher levels of interleukins, suggesting that these treatments have an immunohistochemical profile favorable to recovery and are potential therapeutic strategies.

Local administration of IL-4 or IL-13 may represent a beneficial approach for accelerating tendon healing without compromising systemic immunity.

Ongoing and future studies may validate IL-4 and/or IL-13 as promising therapeutic targets for tendon injuries due to their ability to promote tenocyte proliferation and differentiation. These beneficial effects could be further enhanced using scaffolds, such as the amniotic membrane, which support cell growth and the reconstruction of various tissues, including tendons.

Although the combination of amniotic membrane and stem cells demonstrated promising biological effects in this experimental model, several challenges must be considered for future clinical translation. First, the sourcing, expansion, and quality control of mesenchymal stem cells require strict regulatory oversight, including standardized protocols to ensure cell viability, phenotypic stability, and safety. Second, the preparation, sterilization, and storage of human amniotic membrane must be highly standardized to guarantee consistency between batches, which remains a logistical and regulatory challenge for large-scale clinical use. Additionally, although the amniotic membrane is known to have low immunogenicity, the risk of immune reactions and transmission of infectious agents cannot be entirely excluded and requires rigorous donor screening and processing. These aspects highlight that, while biologically promising, this therapeutic approach still requires further optimization, larger-animal studies, and controlled clinical trials before it can be considered a feasible option for human application.

## Figures and Tables

**Table 1:** Values of type I and type III collagen.

	Group	*N*	Mean	Standard Deviation	Median	Minimum	Maximum	*p* *
Col. I (%)	SC	10	84.3	18.2	90.2	37.2	97.8	
	SC + AM	8	82.0	8.4	83.1	66.3	95.3	
	AM	10	80.3	12.0	83.2	55.6	93.7	
	C	9	88.4	12.1	93.6	68.0	98.0	0.147
Col. III (%)	SC	10	15.7	18.2	9.8	2.2	62.8	
	SC + AM	8	18.0	8.4	16.9	4.7	33.7	
	AM	10	19.7	12.0	16.8	6.3	44.4	
	C	9	11.6	12.1	6.4	2.0	32.0	0.147

Kruskal–Wallis test for statistical analysis of the percentage of type I and III collagen in the groups. * *p* < 0.05 shows statistical significance.

**Table 2:** Final number of each group regarding immunohistochemical analysis.

Variable	Group	*N*
IL-4	SC	10
	SC + AM	7
	AM	9
	C	7
IL-6	SC	6
	SC + AM	4
	AM	8
	C	4
IL-13	SC	8
	SC + AM	6
	AM	9
	C	7

Final *n* of each group for immunohistochemical staining.

**Table 3:** Results of immunohistochemical analysis.

Variable	Group	*N*	Mean	Standard Deviation	Median	Minimum	Maximum	*p* *
IL-6	SC	6	0.18	0.11	0.17	0.06	0.38	
	SC + AM	4	0.93	0.83	0.77	0.24	1.94	
	AM	8	0.22	0.15	0.15	0.08	0.53	
	C	4	0.44	0.26	0.46	0.15	0.70	0.084
IL-13	SC	8	3.9	2.9	3.2	1.3	10.5	
	SC + AM	6	13.6	9.0	11.4	5.6	27.8	
	AM	9	6.8	4.2	6.0	2.0	16.4	
	C	7	2.7	2.5	1.5	0.5	7.3	0.004
IL-4	SC	10	9,8	2.7	8.6	7.0	13.0	
	SC + AM	7	14.9	7.2	13.0	9.6	30.6	
	AM	9	9.9	5.8	9.8	3.0	23.0	
	C	7	6.6	2.5	6.0	2.9	10.5	0.011

Results from the presence of interleukins among the groups. Kruskal–Wallis test for statistical analysis. * *p* < 0.05 shows statistical significance (highlighted in red).

**Table 4:** Comparison of IL-4 and IL-13 between groups.

Compared Groups	IL-13	IL-4
SC × C	1	0.489
SC × AM	0.733	1
SC × SC + AM	0.060	0.381
C × AM	0.101	0.985
C × SC + AM	0.005	0.005
AM × SC + AM	1	0.200

Comparison of percentages of IL-4 and IL-13 between groups. Dunn’s post hoc test. *p* < 0.05 shows statistical significance (highlighted in red).

**Table 5:** Correlation between variables with a significant difference.

Group	Variables Analyzed	*n*	Spearman’s Correlation Coefficient	*p*-Value
MA	IL-4 × collagen I (%)	9	−0.65	0.058
	IL-4 × collagen III (%)	9	0.65	0.058
All	IL-13 × collagen I (%)	30	−0.35	0.057
	IL-13 × collagen III (%)	30	0.35	0.057

Spearman’s correlation coefficient. *p* < 0.05 shows statistical significance.

## Data Availability

The original contributions presented in this study are included in the article. Further inquiries can be directed to the corresponding author.

## Appendix A

**Table A1:** Picrosirius Red data analysis.

Group	Animal	% Col. Type I	% Col. Type III
SC	1	88.67228	11.32772
	2	94.68957	5.310428
	3	94.67009	5.329908
	4	71.99287	28.00713
	5	96.78912	3.210885
	6	97.76461	2.235387
	7	85.19644	14.80356
	8	91.76752	8.232476
	9	83.73942	16.26058
	10	37.24898	62.75102
AM + ST	1	95.25195	4.748048
	2	75.68604	24.31396
	3	83.30736	16.69264
	4	-	-
	5	85.80346	14.19654
	6	66.34731	33.65269
	7	-	-
	8	82.80798	17.19202
	9	81.30873	18.69127
	10	85.55754	14.44246
AM	1	93.74026	6.259735
	2	84.39343	15.60657
	3	82.02189	17.97811
	4	80.07587	19.92413
	5	90.08762	9.91238
	6	87.41478	12.58522
	7	73.04669	26.95331
	8	66.41677	33.58323
	9	55.63869	44.36131
	10	89.67368	10.32632
C (Control)	1	68.02493	31.97508
	2	97.72878	2.271217
	3	68.59478	31.40522
	4	97.01947	2.980529
	5	90.97798	9.022016
	6	96.26597	3.734033
	7	93.64437	6.355624
	8	97.96367	2.036329
	9	85.24229	14.75771
	10	-	-

## Appendix B

**Table A2:** Immunohistochemical data analysis.

Group	Animal	IL-4	IL-6	IL-13
Sc	1	12.6851	0.09289	1.26152
	2	7.08174	0.37515	
	3	7.71709		2.55785
	4	8.97251	0.05649	2.39723
	5	6.95885		3.93043
	6	12.5989	0.22803	2.54793
	7	7.68494		
	8	12.9734		3.78649
	9	8.27816	0.14316	10.5361
	10	12.9734	0.19234	4.18266
SC + AM	1	13.9265		27.8312
	2	13.0249	1.93656	
	3	11.5018		19.8547
	4	30.5643	0.25865	7.75376
	5			
	6	10.2099		15.0871
	7	9.64392	0.24008	5.59404
	8	15.1371	1.28579	5.70805
AM	1			
	2	10.0641	0.53462	7.75376
	3	6.86066		6.01953
	4	22.988	0.30739	8.3792
	5	7.11552	0.1392	4.30523
	6	5.60763	0.07892	3.03288
	7	13.5096	0.16221	1.98716
	8	9.81983	0.11303	7.86464
	9	10.1212	0.1319	5.00698
	10	2.97585	0.32952	16.3635
C	1			
	2	2.8693	0.15288	0.51087
	3	7.73207		5.13824
	4	10.5307	0.28865	1.49216
	5	8.21829	0.69733	1.19455
	6			
	7	6.01073	0.63131	7.3027
	8	4.98184		1.2504
	9	5.5838		2.10399
